# Thyroid Diseases and Breast Cancer

**DOI:** 10.3390/jpm12020156

**Published:** 2022-01-25

**Authors:** Enke Baldini, Augusto Lauro, Domenico Tripodi, Daniele Pironi, Maria Ida Amabile, Iulia Catalina Ferent, Eleonora Lori, Federica Gagliardi, Maria Irene Bellini, Flavio Forte, Patrizia Pacini, Vito Cantisani, Vito D’Andrea, Salvatore Sorrenti, Salvatore Ulisse

**Affiliations:** 1 Department of Surgical Sciences, Sapienza University of Rome, 00161 Rome, Italy; enke.baldini@uniroma1.it (E.B.); augusto.lauro@uniroma1.it (A.L.); domenico.tripodi@uniroma1.it (D.T.); daniele.pironi@uniroma1.it (D.P.); mariaida.amabile@uniroma1.it (M.I.A.); iulia.ferent@uniroma1.it (I.C.F.); eleonora.lori@uniroma1.it (E.L.); federica.gagliardi@uniroma1.it (F.G.); m.irene.bellini@gmail.com (M.I.B.); vito.dandrea@uniroma1.it (V.D.); salvatore.sorrenti@uniroma1.it (S.S.); 2 Department of Urology, M.G. Vannini Hospital, 00177 Rome, Italy; flavioforte@hotmail.com; 3 Department of Radiological, Anatomopathological and Oncological Sciences, Sapienza University of Rome, 00161 Rome, Italy; patrizia.pacini@uniroma1.it (P.P.); vito.cantisani@uniroma1.it (V.C.)

**Keywords:** thyroid disease, breast cancer, etiology, epidemiology, extra-thyroidal malignancies

## Abstract

Epidemiological studies aimed at defining the association of thyroid diseases with extra-thyroidal malignancies (EM) have aroused considerable interest in the possibility of revealing common genetic and environmental factors underlying disease etiology and progression. Over the years, multiple lines of evidence indicated a significant relationship between thyroid carcinomas and other primary EM, especially breast cancer. For the latter, a prominent association was also found with benign thyroid diseases. In particular, a meta-analysis revealed an increased risk of breast cancer in patients with autoimmune thyroiditis, and our recent work demonstrated that the odds ratio (OR) for breast cancer was raised in both thyroid autoantibody-positive and -negative patients. However, the OR was significantly lower for thyroid autoantibody-positive patients compared to the negative ones. This is in agreement with findings showing that the development of thyroid autoimmunity in cancer patients receiving immunotherapy is associated with better outcome and supports clinical evidence that breast cancer patients with thyroid autoimmunity have longer disease-free interval and overall survival. These results seem to suggest that factors other than oncologic treatments may play a role in the initiation and progression of a second primary malignancy. The molecular links between thyroid autoimmunity and breast cancer remain, however, unidentified, and different hypotheses have been proposed. Here, we will review the epidemiological, clinical, and experimental data relating thyroid diseases and breast cancer, as well as the possible hormonal and molecular mechanisms underlying such associations.

## 1. Introduction

**Figure 1:**
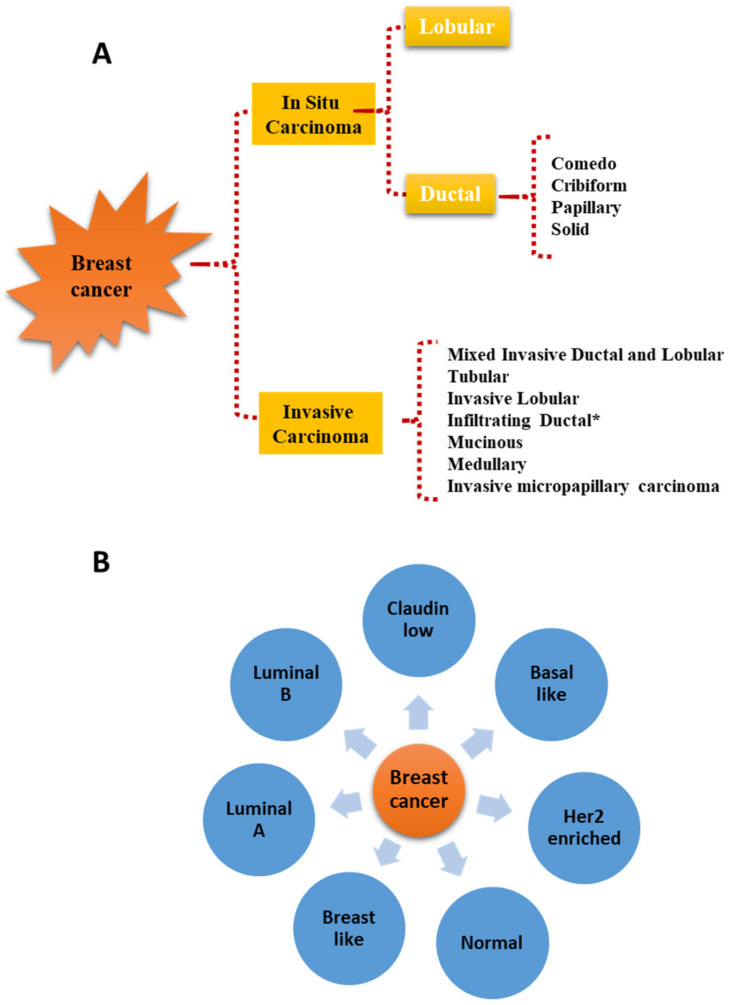
Histological (**A**) and molecular (**B**) classification of breast cancer. * Infiltrating ductal carcinomas evaluated on the basis of nuclear morphology, glandular/tubule formation, and mitotic index are further sub-classified in well-differentiated, moderately differentiated, and poorly differentiated carcinomas.

The association of either benign or malignant thyroid diseases (TD) with extra-thyroidal malignancies (EM) has been highlighted in several epidemiological studies, and whether a causal relationship exists between them has been a matter of debate over the last decades. Primarily, such connections have generated much interest around the possibility of identifying common genetic and environmental factors responsible for the etiology and progression of these diseases. In particular, a number of different reports have described the association between thyroid cancers and other primary EM, including breast cancer (BC) [1,2,3,4]. This has led to the hypothesis that the long-term carcinogenic effects of anticancer treatments could be responsible for a second primary cancer. In this area, several researchers evaluated whether the ^131^I therapy administered to thyroid cancer patients could represent the main cause of a succeeding primary EM. Some of them indicated a 30–42% increased risk of primary EM following ^131^I exposure, but others did not recognize such correlation. Similarly, studies aimed at clarifying whether anticancer treatments of EM, in particular external beam radiations, may cause subsequent primary thyroid cancers have produced conflicting results. On the other hand, a significant association between benign TD and BC was also shown to occur, which seems to suggest that factors other than oncologic treatment may play a role in the initiation and progression of second malignancies [5,6,7,8]. It has to be mentioned, however, that some biases in the epidemiological studies reporting associations between TD and BC could exist: (i) TD and BC are very common diseases increasing with age in the female population, which make difficult to discern a real link from a chance association; (ii) the majority of studies examining the association of TD with BC are retrospective or cross-sectional and thus more susceptible to biases compared to prospective studies; (iii) both BC and TD are heterogeneous diseases, and only in a minority of reports, the different BC characteristics such as histology and/or molecular subtypes (Figure 1) and/or TD types were examined.

In the present manuscript, we will review the epidemiological, experimental, and clinical evidence relating benign TD and BC, as well as the potential molecular mechanisms underlying their associations. In particular, approaching the possible role(s) of thyroid gland dysfunctions in promoting breast cancer, we will take into consideration the effects of deregulated thyrotropin (TSH), thyroid hormone (TH), and prolactin (PRL) levels, as well as the effects of thyroid-specific autoantibodies on BC progression. 

## 2. Association of Thyroid Diseases with Breast Cancer: A Historical View

The first clinical evidence of TD and BC association was reported in 1896, when Beatson described the beneficial effects of bilateral oophorectomy and thyroid extract administration in metastatic BC patients [9]. In the 1950s, a number of clinical and experimental results suggested the presence of a causal relationship between hypothyroidism and BC [10,11,12,13,14,15]. These included the demographic coincidence of the two diseases, the high incidence of goiter or thyroid atrophy in BC patients, the observation that TH treatment reduced the number of recurrences in mastectomized BC patients, and the ability of thyroxine treatment to protect experimental animals against tumor progression, whereas an induced hypothyroid state enhanced tumor growth [10,11,12,13,14,15]. However, few years later the association between hypothyroidism and BC was put in doubt [16,17,18]. In particular, epidemiological data showed that despite a dramatic decrease in the state of Michigan of goiter prevalence, from 38.6%, recorded in 1924, to 1.4%, recorded in 1951, the cancer death rate from BC remained unaltered [16,17]. Some studies even reported that breast and other cancers were less frequently encountered in hypothyroid patients [10,19,20]. Backwinkel and Jackson argued that the high incidence of thyroid deficiency in BC patients does not prove a causal relationship but rather reflects the overall incidence of goiter in a given population or area [18]. The same authors, following the analysis of a larger series of patients, documented that the mean age at diagnosis of hypothyroid BC patients was not much different from that of euthyroid ones, and similarly the survival time of BC patients did not differ between euthyroid and hypothyroid individuals [18]. However, when they considered metastatic BC, they found that hypothyroid patients had significantly lower survival [18]. Likewise, Moossa and colleagues showed that, despite the lack of association between the development of BC and hypothyroidism, patients with TD had a shorter survival [21]. At that time, different clinical findings started to indicate a promoter role of thyrotropin (TSH) in TC growth and metastatization [22,23,24]. This raised the question of whether the increased level of TSH or other trophic hormone(s) released by the pituitary in hypothyroid subjects, rather than the lack of the thyroid hormone itself, unleashed the metastatic spread of BC [18]. Eskin and colleagues, investigating the role of iodine deficiency in the pathogenesis of BC, established a relationship between iodine shortage and breast dysplasia and neoplasia, suggesting that TSH could play an important role in the induction of breast dysplasia in conditions of both iodine deficit and primary hypothyroidism [25,26]. In those years, the ability of thyrotropin-releasing hormone (TRH) to stimulate the release of prolactin (PRL) in addition to TSH was also recognized, thus explaining the presence of high serum levels of PRL and the occurrence of galactorrhea in hypothyroid patients [27,28]. Specific PRL receptors were identified in both normal breast and BC tissues, and the potential involvement of PRL in the pathogenesis of BC emerged from several experiments in animals and clinical observations [29,30]. In addition, Mittra and colleagues hypothesized that circulating TH could regulate the mammotropic effects of PRL and that the increase of PRL activity occurring in the hypothyroidism condition might lead to dysplasia and eventually neoplasia of breast cells [31,32,33]. Another possible way in which TH could affect BC was proposed in the 1960s, when the effects of TH on estrogen and androgen metabolism as well as on the plasma levels of sex hormone binding globulin (SHBG) began to be recognized [34,35,36,37]. Following the diffusion of radioimmunoassays, in the 1970s, some studies described the presence of higher TSH and lower triiodothyronine (T_3_) plasma levels in early and advanced BC patients compared to control women [38,39,40]. A single report showed that the free thyroxine level in BC patients was significantly lower than in healthy individuals and inversely correlated with tumor differentiation [41]. However, other studies did not confirm these findings [42,43]. Interestingly, Shering and colleagues pointed out that while the prevalence of hyper- and hypothyroidism in patients with BC and healthy women was similar, the presence of non-toxic goiter was more than twice as common and thyroid volume was significantly higher in BC patients compared to controls [43]. This led to the hypothesis that also subclinical hypothyroidism could be of some importance in the etiology of BC [32]. Actually, Adami and colleagues reported that the prevalence of TD and the need for thyroxine (T_4_) treatment did not differ between control women and BC patients, but the latter had a higher TSH mean value and a lower T_3_ mean value, despite no variations being observed in T_4_ levels [44]. Such a pattern was found in several non-thyroidal diseases, so it was suggested that it might represent a secondary and probably extrathyroidal metabolic change, most likely due to altered T_4_ peripheral conversion [44]. In the 1960s, the first study appeared describing a high incidence of BC among TC patients [45]. In this work, Chalstrey and Benjamin documented the occurrence of BC in 8 out of 92 female patients affected by TC. In three cases, BC preceded TC, in three cases, the opposite occurred, and in two cases, BC and TC were diagnosed at the same time [45]. In 1976, Mittra and colleagues reported the first data on the prevalence of thyroid autoantibodies in Japanese and British BC patients [46]. These authors found that the incidence of thyroid microsomal or thyroglobulin autoantibodies measured by means of immunofluorescence and hemagglutination tests, respectively, was two to three times higher in healthy British women compared to healthy Japanese women. However, no differences in the prevalence of thyroid autoimmunity between BC patients and their relative control groups in either group was recorded [46].

**Figure 2:**
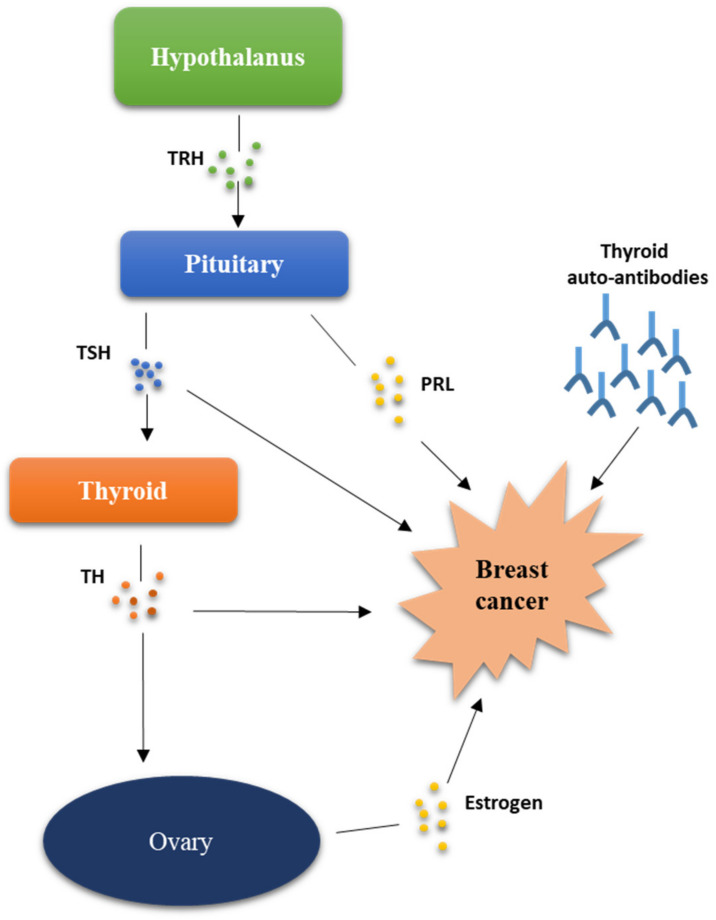
Influence of the hypothalamic–pituitary–thyroid axis on breast cancer progression. Thyrotropin-releasing hormone (TRH); thyrotropin (TSH); prolactin (PRL); thyroid hormone (TH).

In conclusion, from all the information acquired so far, it appears that alterations of the hypothalamus–pituitary–thyroid axis could affect BC progression in several ways, as schematically depicted in Figure 2. In the next paragraphs, we will attempt to address each of these aspects in the light of the most recent epidemiological, clinical, and experimental findings.

## 3. Thyroid Disease, Prolactin, and Breast Cancer

During pregnancy, increased estrogen levels stimulate lactotroph cell proliferation, leading to increased PRL secretion by the pituitary. The released hormone, along with estradiol, progesterone, placental lactogen, insulin, and cortisol, stimulates mammary gland (MG) growth. In this period, the ability of PRL to induce milk synthesis (lactogenic effect) is inhibited by the high plasma concentration of estrogen. After childbirth, the estrogen level falls, allowing the lactogenic effect of PRL to take place. During lactation, short-term peaks in PRL secretion occur after nipple stimulation by the suckling infant through a neuro-humoral axis [47,48]. At the cellular level, the action of PRL is mediated by the PRL receptor (PRL-R), a single transmembrane protein belonging to the cytokine/hematopoietin receptor superfamily [47,49]. The PRL-R gene is located on chromosome 5 and is expressed in several tissues including gonads, uterus, breast, liver, kidney, cells of the immune system, and others [47,49]. The PRL-R gene contains 11 exons including 5 alternatives first exons, E11 to E15, each having its own tissue-specific promoter [47,49]. Thus, the pleiotropic effects of PRL may also arise from the tissue-specific expression of PRL-R variants. Upon ligand activation, PRL-R has been shown to activate the intracellular JAK–STAT and mitogen-activated protein kinases (MAPK) signaling pathways [50]. Despite the first evidence being acquired more than 40 years ago, whether PRL and its cognate receptor play a role in BC initiation and progression is still an area of active debate. In addition to the earlier observations previously mentioned, recent works in vitro and in experimental animals appear to confirm a role of PRL in promoting BC [49,50,51,52,53,54]. In particular, the expression of PRL and its receptor has been detected at both the mRNA and protein levels in more than 90% of invasive human BC, suggesting the presence of autocrine/paracrine effects of PRL within BC tissues [51]. Studies in BC-derived cell lines demonstrated the mitogenic action of PRL [50], and activation of PRL-R has been shown to be sufficient and required for the induction of mammary carcinoma in mice [52,53,54]. In humans, however, epidemiological studies focused on the promoting role of PRL in BC have produced inconsistent results [49]. In fact, large prospective cohort investigations estimated a significantly increased BC risk in postmenopausal women having serum PRL levels in the top quartile of the normal range compared to age-paired women with PRL serum levels in the bottom quartile [55,56,57,58]. Furthermore, this risk was stronger for women with estrogen receptor (ER)-positive tumors [56,57,58]. On the other hand, two epidemiological studies concerning women with hyperprolactinemia failed to demonstrate in these patients an increased risk of BC [59,60]. To this regard, it could be speculated that locally produced PRL, rather than circulating PRL released by the pituitary, has a role in supporting BC growth. On the whole, the above evidence suggests that PRL, either of pituitary origin or not, likely boosts BC progression. 

Deregulated thyroid functions could affect PRL action in promoting BC by at least two different ways. The first one, as mentioned above, implies TRH overstimulation of pituitary PRL secretion in hypothyroidism conditions [27,31,32,33]; the second one relies on the reported ability of TH to enhance PRL-induced promotion of lobuloalveolar development in breast organ cultures [50,61]. However, the underlying molecular mechanism(s) of such effect remain(s) to be determined.

## 4. Thyrotropin (TSH), Graves’ Disease, and Breast Cancer

Besides its well-known and characterized role in the regulation of thyroid gland development and thyrocytes differentiation and proliferation, TSH is thought to modulate the function of several extrathyroidal tissues. In fact, a growing number of experimental findings evidenced the expression of TSH receptor (TSH-R) in several non-thyroid cells, including murine and human normal and BC tissues [62,63,64,65,66,67]. 

In thyrocytes, upon ligand binding, TSH-R interacts with Gs proteins leading to the stimulation of adenylate cyclase (cAMP) formation and protein kinase A (PKA) activation. It also interacts with Gq proteins, resulting in phospholipase C (PLC) stimulation, intracellular Ca++ influx, and increased protein kinase C (PKC) activity. In addition, TSH-R can signal through alternate pathways including p38, p42/44 MAPK, and phosphoinositide 3-kinase (PI3K) [62]. However, the mitogenic action of TSH in thyrocytes seems to depend on the presence of additional growth factors, such as insulin or IGF-1 [68,69]. Moreover, TSH can indirectly stimulate thyrocyte proliferation by increasing the expression of autocrine growth factors or their cognate receptors [68,69]. Clinical observations showing that high serum TSH concentrations are associated with an increased risk of thyroid cancer (TC) and a reduced disease-specific survival have led to consider TSH a tumor promoter in differentiated TC (DTC) patients [70,71,72]. This effect is presumed to arise from the excessive intracellular activation of pathways associated with the proliferative response [55]. Therefore, a mainstay of the clinical management of these patients is the administration of exogenous L-thyroxine (L-T_4_) to reduce or suppress serum TSH levels in patients at low or high risk for DTC recurrences, respectively [73]. All together, these observations suggest that TSH may represent a promoting factor also for EM whose cancer cells express functional TSH-R. For instance, Ellerhorst and colleagues demonstrated the presence of TSH-R in cutaneous melanocytic lesions, including nevi, dysplastic nevi, and melanomas, with the highest expression found in malignant and pre-malignant lesions [74]. In these cells, TSH at physiological concentrations was capable of inducing cAMP formation and MAPK activation, as well as malignant cell proliferation [74]. 

Govindaraj and colleagues observed the expression of TSH-R mRNA and protein in normal and cancer breast cells, which was significantly higher in tumor tissues compared to normal breast tissues [67]. The observation made by Shi and colleagues in lactating mice is also of interest [66]. They found that breast tissue expresses a TSH-R characterized by a 173 aa deletion in the extracellular domain, causing a less efficient binding to TSH. This could explain the lower expression of natrium iodide symporter (NIS) in lactating breast compared to thyroid tissue [66]. Thus, even if further studies are needed, in vitro results converge to point that TSH may have a role in BC progression, and actually there is clinical evidence supporting this idea [5,75,76,77,78,79]. In particular, a strong association of BC with Graves’ disease, caused by autoantibodies to the TSH-R, but not with non-immune hyperthyroidism has been demonstrated [75]. A population-based cohort study in Sweden comprising 18,156 hospitalized Graves’ disease patients detected an increased risk of BC in this group [5]. Similarly, a study examining 5025 cases of Graves’ disease in Taiwan reported a hazard ratio for developing BC of about 1.6 [76]. 

To date, a major gap in this knowledge is represented by the lack of in vitro studies on the function(s) of TSH-R in BC cells, which could shed light on the signal transduction pathways actually involved in the proliferative stimulus in this cell type.

## 5. Thyroid Hormones and Breast Cancer

### 5.1. Thyroid Hormone Secretion and Mechanisms of Action in Target Tissues

Thyroid hormones are major regulators of growth and development as well as of a number of homeostatic functions in adults, including energy and heat production [80]. Thyroid follicular cells produce two THs, 3,5,3′,5′-L-tetraiodothyronine (thyroxine, T_4_) and 3,5,3′-L-triiodothyronine (T_3_). Once secreted into the blood, THs are carried by three major transport proteins: thyroxine-binding globulin (TBG), thyroxine-binding prealbumin (TBPA or transthyretin), and albumin. The thyroid gland produces about 100 nmol of T_4_ and 5 nmol of T_3_ every day to maintain a serum total T_4_ concentration of about 103 nmol/L and a total T_3_ concentration of about 1.8 nmol/L [80]. Only 0.04% of total T_4_ (about 19 pmol/L) and 0.4% of total T_3_ (about 4.3 pmol/L) circulate in free form and are responsible for the hormonal effects on target tissues [81]. The free THs may act on target cells by two distinct mechanisms: genomic and non-genomic [81,82]. The classical genomic action starts when THs enter the target cells through several plasma membrane transporters, e.g., the monocarboxylate transporters MCT8 and MCT10, the organic anion transporters OATP1 and OATP3, and the L-type amino acid transporter LAT [83]. Once in the cytoplasm, T_4_ is deiodinated by deiodinases 1 (D1) or 2 (D2) to form T_3_, which binds TH nuclear receptor (THR) with greater affinity compared to T_4_ [80]. THRs belong to the nuclear receptor superfamily and act as ligand-dependent transcription factors that bind to specific DNA sequences within promoter regions, known as thyroid responsive elements (TRE), and induce or repress the transcription of downstream target genes [84,85]. Two THR proteins have been identified, THRα and THRβ, encoded by the THRA gene, located on chromosome 17, and the THRΒ gene, located on chromosome 3 [86,87,88]. From the THRA gene, three different transcripts are generated, THRα1, THRα2, and THRα3, of which only THRα1 is able to bind T_3_ [89,90]. Compared to THRα1, THRα2 and THRα3 proteins differ in sequence and in the length of the C-terminal region. The truncated THRα2 and THRα3 receptors can heterodimerize with the full-length receptor and antagonize T_3_-mediated transcriptional regulation [89,90]. The THRΒ gene provides two receptor isoforms differing in their tissue distribution, THRβ1 and THRβ2, both of which bind T_3_ [85].

The second mechanism of THs action, by which THs may elicit rapid cellular responses, is initiated at the plasma membrane, where both T_4_ and T_3_ may attach to specific regions present in the integrin αvβ3 [81,82,91]. In particular, the latter contains two TH binding sites, termed S1 and S2, of which S1 binds only T_3_ at physiological serum concentrations and leads to the intracellular activation of the PI3K, while S2 binds T_4_ and, to a lesser extent, T_3_, inducing the intracellular activation of extracellular signal-regulated kinases (ERK) 1 and 2 [81,82,92]. 

### 5.2. Thyroid Hormones and Breast Development and Cancer

THs have been suggested to play a role, along with other hormones (i.e., PRL, estrogen, progesterone, insulin, growth hormone, and adrenal steroids) in normal breast growth and development [93,94]. In particular, high-affinity binding sites for T_3_ have been identified in the mammary gland and are thought to modulate, upon ligand attachment, ductal branching, alveolar budding, and lobules enlargement [95,96,97,98]. Moreover, in view of their ability to activate PRL plasma membrane receptors and to enhance casein synthesis induced by PRL, THs are considered lactopoietic [93,94,99,100].

The relevance of ERs overexpression during BC progression is well recognized and is such that the most commonly used drugs, i.e., tamoxifen, fulvestrant, and aromatase inhibitors, are aimed at reducing estrogen levels or blocking ER signaling [101,102]. The extensive use of these drugs in the adjuvant therapy of BC is held accountable for the reduced mortality of patients [103,104,105,106,107]. Experimental evidence suggests that TH could support the estrogen-dependent proliferation of BC cells in several ways: (i) TH may increase the expression of estrogen receptors (ERs) [108,109]; (ii) TRE and the ER response element (ERE) share an identical half-site, and THRs have been shown to bind also to ERE [110]; (iii) thyroxine, through the αvβ3 integrin receptor, may activate MAPK signaling and the phosphorylation of the nuclear ERα [111]. This phosphorylation affects ER ability to interact with chromatin, to recruit coregulators, and to modulate gene expression even in the absence of estrogen [111,112,113]. In addition, through integrin receptor signaling, THs were found to favor the proliferation of BC cells lacking ER [114,115]. Other than by their effect on the cell cycle, THs have been shown to prompt BC progression by stimulating aerobic glycolysis (Warburg effect), a hallmark of malignant cells [116,117]; BC cell migration and invasion [116,118]; the expression of Programmed Cell Death Ligand 1 (PD-L1), thus preventing the immune destruction of BC cells [116,119]. These observations are in agreement with a number of recent epidemiological investigations indicating that THs may support BC growth in both pre- and postmenopausal women and with clinical data showing that hypothyroidism may have protective effects by reducing the incidence and progression of BC [6,91,120,121,122,123,124,125,126,127,128,129,130,131,132,133,134,135,136,137,138,139,140,141]. It is worth considering that T_4_ maximally stimulates αvβ3 at physiological free-T_4_ concentrations, while supraphysiological free-T_3_ concentrations are required to induce cell proliferation via this receptor [113,142,143]. Notably, in a compassionate study comprising patients with far-advanced solid tumors, including BC, Hercbergs and colleagues reported that medically induced euthyroid hypothyroxinemia (pharmacological elimination of T_4_ and replacement by T_3_) extended patient’s survival [113,144]. This represents an attractive new therapeutic approach that deserves larger clinical studies to be confirmed. 

Nonetheless, it should be taken into account that, while αvβ3 receptors are thought to mediate most of the tumor-promoting effects of THs in BC cells, nuclear THRs appear to play oncosuppressive functions in BC as well as in other solid tumors [145]. The expression of THRs has been documented in BC tissues [146,147]. In particular, Silva and colleagues demonstrated the presence of THRα1 and THRβ1, but not of THRβ2, at both protein and mRNA levels in 70 sporadic BC tissues [146]. However, the loss of the THRΒ gene following truncation or deletion of chromosome 3p, where it is located, or loss of heterozygosity (LOH) and gene rearrangement of the THRA gene have been shown to occur in BC samples [92,145,148]. Somatic mutations of THRs leading to reduced ligand affinity and transcription activity, as well as THRB gene promoter hypermethylation with consequent reduced gene expression, have been also described in BC tissues [145,149,150,151]. The tumor suppressor role of THRΒ has been further validated by Park and colleagues, who overexpressed the THRB gene in the human BC-derived cell line MCF-7, endowed with ER and responsive to estrogen stimulation [152]. In a mouse xenograft model, these MCF-7 cells showed a significantly impaired growth due to reduced proliferation and activation of apoptotic pathways [152].

In conclusion, the imbalance of expression and/or activation between membrane and nuclear TH receptors may have detrimental consequences on BC progression. 

## 6. Autoimmune Thyroid Disease (AITD) and Breast Cancer (BC)

Studies aimed at defining the association between BC and benign TD, in particular AITD, have produced conflicting results causing a long-lasting debate [153,154,155,156,157,158,159,160,161,162,163,164]. In 2002, Sarlis and colleagues performed a meta-analysis of 13 articles published over the previous 50 years including 14,226 women [153]. The authors failed to demonstrate any association between Hashimoto thyroiditis (HT) and BC [153]. Ten years later, Hardefeldt and colleagues accomplished a meta-analysis comprising 28 studies and showed the presence of a higher risk of BC in patients with AITD [7]. In addition, their results testified an increased BC risk associated with the presence of anti-thyroid antibodies and goiter, with Odds Ratios (OR) of 2.92, and 2.26, respectively [7]. The latter data were confirmed in 2020 by Pan and colleagues by means of a meta-analysis on 11 different studies [8]. The authors could establish that patients with BC had higher titers of anti-thyroid peroxidase antibodies (TPOAb) and anti-thyroglobulin antibodies (TgAb) compared to a non-breast disease control group [8]. Similarly, in a very recent meta-analysis involving 21 studies, Chen and colleagues identified TgAb and TPOAb as significantly associated with an increased risk of BC [6]. In our Institute, we analyzed the prevalence of EM in 6386 female patients affected by different TD and we found that a number of EM were associated with TD [79,154]. The EM most frequently recorded was BC (OR 3.94), followed by colorectal (OR 2.18), melanoma (OR 6.71), hematological (OR 8.57), uterus (OR 2.52), kidney (OR 3.40), and ovary (OR 2.62) neoplasms. By age-matched analysis, we observed that the risk of EM was maximal in the age group 0–44 years (OR 11.28), remaining lower but significantly higher than that observed in the general population in the 45–59 and 60–74-years groups [154]. We also showed that when TD patients were dichotomized based on the presence or the absence of TgAb and/or TPOAb, both groups had a higher risk of BC compared to the general population, but the risk was significantly lower in autoantibody-positive patients [79,154]. This finding suggests that amongst TD patients, the presence of thyroid autoantibodies may have a partial protective effect against BC. The latter hypothesis is in agreement with an earlier observation by Smyth and colleagues on TPOAb-positive BC patients, who had a significantly better disease-free and overall survival compared to patients who were TPOAb-negative [155]. In this context, the study by Weijl and colleagues reporting the occurrence of hypothyroidism and anti-thyroid antibodies in patients affected by different types of cancer and undergoing immunotherapy with interleukin-2 is of some interest [156]. They found that the preexistence or development of thyroid autoantibodies-related hypothyroidism was associated with a favorable response to immunotherapy [156]. Similar observations were reported by Franzke and colleagues, who observed that autoimmunity caused by IL-2 and IFN-α2 treatment predicted long-term survival in patients affected by metastatic renal cell cancer [157]. To explain the protective role of thyroid autoantibodies, it has been proposed that cell-mediated cytotoxicity elicited by these antibodies against shared antigens may affect the thyroid gland as well as the tumor [158,159]. This hypothesis is consistent with the expression of NIS and TPO noticed in breast tissues [158,160]. Despite this evidence, however, further prospective large case studies should be undertaken to definitely prove the protective role of thyroid antibodies in BC cancer progression.

## 7. Conclusions

The impact of thyroid axis dysfunctions on BC progression has been a matter of debate for more than a century, and still today many controversies exist. The available information strongly suggests that TD may affect BC progression in several ways, through (i) altered plasma levels of TSH and THs or production of specific thyroid autoantibodies; (ii) dysregulation of PRL secretion due to hypothyroidism; (iii) alterations in THs responsiveness of BC cells. Thus, different hormonal and molecular players should be taken into consideration in every single patient, when analyzing the association between TD and BC. This knowledge will likely shed light on the potential pathogenic links between TD and BC, possibly allowing a more personalized clinical management of these patients. 

## Data Availability

Not applicable.

## References

[1] Bhatti P., Veiga L.H., Ronckers C.M., Sigurdson A.J., Stovall M., Smith S.A., Weathers R., Leisenring W., Mertens A.C., Hammond S. (2010). Risk of second primary thyroid cancer after radiotherapy for a childhood cancer in a large cohort study: An update from the childhood cancer survivor study. Radiat. Res..

[2] Trinh L.N., Crawford A.R., Hussein M.H., Zerfaoui M., Toraih E.A., Randolph G.W., Kandil E. (2021). Deciphering the risk of developing second primary thyroid cancer following a primary malignancy—Who is at the greatest risk?. Cancers.

[3] Zhang Y., Liang J., Li H., Cong H., Lin Y. (2016). Risk of second primary breast cancer after radioactive iodine treatment in thyroid cancer: A systematic review and meta-analysis. Nucl. Med. Commun..

[4] Mahmood S., Vu K., Tai P., Joseph K., Koul R., Dubey A., Yu E. (2015). Radiation-induced second malignancies. Anticancer Res..

[5] Shu X., Ji J., Li X., Sundquist J., Sundquist K., Hemminki K. (2010). Cancer risk in patients hospitalised for Graves’ disease: A population-based cohort study in Sweden. Br. J. Cancer.

[6] Chen S., Wu F., Hai R., You Q., Xie L., Shu L., Zhou X. (2021). Thyroid disease is associated with increased risk of breast cancer: A systemic review and meta-analysis. Gland. Surg..

[7] Hardefeldt P.J., Eslick G.D., Edirimanne S. (2012). Benign thyroid disease is associated with breast cancer: A meta-analysis. Breast Cancer Res. Treat..

[8] Pan X.F., Ma Y.J., Tang Y., Yu M.M., Wang H., Fan Y.R. (2020). Breast cancer populations may have an increased prevalence of thyroglobulin antibody and thyroid peroxidase antibody: A systematic review and meta-analysis. Breast Cancer.

[9] Beatson G.T. (1896). On the treatment of inoperable cases of carcinoma of the mamma: Suggestions for a new method of treatment, with illustrative cases. Trans. Med. Chir. Soc. Edinb..

[10] Finley J.W., Bogardus G.M. (1960). Breast cancer and thyroid disease. Q. Rev. Surg. Obstet. Gynecol..

[11] Loeser A.A. (1954). A new therapy for the prevention of post-operative recurrences in genital and breast cancer; a six-years study of prophylactic thyroid treatment. BMJ.

[12] Sommers S.C. (1955). Endocrine abnormalities in women with breast cancer. Lab. Investig..

[13] Edelstyn G.A., Lyons A.R., Welbourn R.B. (1958). Thyroid function in patients with mammary cancer. Lancet.

[14] Spencer J.G.C. (1954). Influence of thyroid in malignant disease. Br. J. Cancer.

[15] Repert R.W. (1952). Breast carcinoma study: Relation to thyroid disease and diabetes. J. Mich. State Med. Soc..

[16] Kimball O.P. (1953). History of prevention of endemic goiter. Bull. World Health Organ..

[17] Pack G.T., Ariel I.M., Pack G.T., Ariel I.M. (1960). Introduction: Classification, Principles of Treatment, and Prognosis of Tumors of Breast. Treatment of Cancer and Allied Diseases.

[18] Backwinkel K., Jackson A.S. (1964). Some features of breast cancer and thyroid deficiency. Cancer.

[19] Levy J., Levy J.A. (1951). Role of hypometabolic state in cancer. Am. Pract Digest Treat..

[20] Humphrey L.J., Swerdlow M. (1964). The relationship of breast disease to thyroid disease. Cancer.

[21] Moossa A.R., Price Evans D.A., Brewer A.C. (1973). Thyroid status and breast cancer: Reappraisal of an old relationship. Ann. R. Coll. Surg. Engl..

[22] Crile G. (1955). Treatment of cancer of thyroid with dessicated thyroid. Clevel. Clin. Quart..

[23] Molnar G.D., Colby M.Y., Woolner L.B. (1963). Demonstration of thyroid-stimulating hormone dependence in case of metastatic carcinoma of thyroid origin. Proc. Staff Meet. Mayo Clin..

[24] Sonenberg M., Brener J. (1964). Inhibitory effects of acetylated thyrotropin on metastatic thyroid cancer. Cancer.

[25] Eskin B.A. (1970). Iodine metabolism and breast cancer. Trans. N.Y. Acad. Sci..

[26] Eskin B.A., Bartuska D.G., Dunn M.R., Jacob G., Dratman M.B. (1967). Mammary gland dysplasia in iodine deficiency. Studies in rats. JAMA.

[27] Edwards C.R.W., Forsyth I.A., Besser G.M. (1971). Amenorrhoea, galactorrhoea, and primary hypothyroidism with high circulating levels of prolactin. Br. Med. J..

[28] Bowers C.Y., Friesen H.G., Hwang P., Guyda H.J., Folkers K. (1971). Prolactin and thyrotropin release in man by synthetic pyroglutamyl-histidyl-prolinamide. Biochem. Biophys. Res. Commun..

[29] Kirschner M.A. (1977). The role of hormones in the etiology of human breast cancer. Cancer.

[30] Salih H., Flax H., Brander W., Hobbs J.R. (1972). Prolactin dependence in human breast cancers. Lancet.

[31] Mittra I. (1974). Mammotropic effect of prolactin enhanced by thyroidectomy. Nature.

[32] Mittra I., Hayward J.L. (1974). Hypothalamic-pituitary-thyroid axis in breast cancer. Lancet.

[33] Mittra I., Hayward J.L., McNeilly A.S. (1974). Hypothalamic-pituitary-prolactin axis in breast cancer. Lancet.

[34] Fishman J., Hellman L., Zumoff B., Callagher T.F. (1962). Influence of thyroid hormone on estrogen metabolism in man. J. Clin. Endocrinol. Metab..

[35] Skovsted L., Hansen J.M., Kristensen M., Christensen L.K. (1966). The androsterone-etiocholanolone excretion ratio in hyper- and hypothyroidism. Acta Med. Scand..

[36] Ruder M., Corvol P., Mahoudeau J.A., Ross G.T., Lipsett M.B. (1971). Effects of induced hyperthyroidism on steroid metabolism in man. J. Clin. Endocrinol. Metab..

[37] Burke C.W., Anderson D.C. (1972). Sex-hormone-binding globulin is an oestrogen amplifier. Nature.

[38] Rose D.P., Davies T.E. (1978). Plasma thyroid-stimulating hormone and thyroxine concentrations in breast cancer. Cancer.

[39] Rose D.P., Davies T.E. (1979). Plasma triiodothyronine concentrations in breast cancer. Cancer.

[40] Perry M., Goldie D.J., Self M. (1978). Thyroid function in patients with breast cancer. Ann. R. Coll. Surg. Engl..

[41] Thomas B.S., Bulbrook R.D., Russell M.J., Hayward J.L., Millis R. (1983). Thyroid function in early breast cancer. Eur. J. Cancer Clin. Oncol..

[42] MacFarlane I.A., Robinson E.L., Bush H., Durning P., Howat J.M.T., Beardwell C.G., Shalet S.M. (1980). Thyroid function in patients with benign and malignant breast disease. Br. J. Cancer.

[43] Shering S.G., Zbar A.P., Moriarty M., McDermott E.W., O’Higgins N.J., Smyth P.P. (1996). Thyroid disorders and breast cancer. Eur. J. Cancer Prev..

[44] Adami H.O., Rimsten A., Thorén L., Vegelius J., Wide L. (1978). Thyroid disease and function in breast cancer patients and non-hospitalized controls evaluated by determination of TSH, T3, rT3 and T4 levels in serum. Acta Chir. Scand..

[45] Chalstrey L.J., Benjamin B. (1966). High incidence of breast cancer in thyroid cancer patients. Br. J. Cancer.

[46] Mittra I., Perrin J., Kumaoka S. (1976). Thyroid and other autoantibodies in British and Japanese women: An epidemiological study of breast cancer. Br. Med. J..

[47] Ignacak A., Kasztelnik M., Sliwa T. (2012). Prolactin—Not only lactotrophin. A new view of the old hormone. J. Physiol. Pharmacol..

[48] Capozzi A., Scambia G., Pontecorvi A., Lello S. (2015). Hyperprolactinemia: Pathophysiology and therapeutic approach. Gynecol. Endocrinol..

[49] Bernard V., Young J., Chanson P., Binart N. (2015). New insight in prolactin: Pathological implications. Nat. Rev. Endocrinol..

[50] Das R., Vonderhaar B.K. (1997). Prolactin as a mitogen in mammary cells. J. Mammary Gland. Biol. Neoplasia.

[51] Reynolds C., Montone K.T., Powell C.M., Tomaszewski J.E., Clevenger C.V. (1997). Expression of prolactin and its receptor in human breast carcinoma. Endocrinology.

[52] Wennbo H., Gebre-Medhin M., Gritli-Linde A., Ohlsson C., Isaksson O.G., Törnell J. (1997). Activation of the prolactin receptor but not the growth hormone receptor is important for induction of mammary tumors in transgenic mice. J. Clin. Investig..

[53] Vomachka A.J., Pratt S.L., Lockefeer J.A., Horseman N.D. (2000). Prolactin gene-disruption arrests mammary gland development and retards T-antigen-induced tumor growth. Oncogene.

[54] Oakes S.R., Robertson F.G., Kench J.G., Gardiner-Garden M., Wand M.P., Green J.E., Ormandy C.J. (2007). Loss of mammary epithelial prolactin receptor delays tumor formation by reducing cell proliferation in low-grade preinvasive lesions. Oncogene.

[55] Hankinson S.E., Willett W.C., Michaud D.S., Manson J.E., Colditz G.A., Longcope C., Rosner B., Speizer F.E. (1999). Plasma prolactin levels and subsequent risk of breast cancer in postmenopausal women. J. Natl. Cancer Inst..

[56] Tworoger S.S., Eliassen A.H., Rosner B., Sluss P., Hankinson S.E. (2004). Plasma prolactin concentrations and risk of postmenopausal breast cancer. Cancer Res..

[57] Tworoger S.S., Eliassen A.H., Sluss P., Hankinson S.E. (2007). A prospective study of plasma prolactin concentrations and risk of premenopausal and postmenopausal breast cancer. J. Clin. Oncol..

[58] Tworoger S.S., Eliassen A.H., Zhang X., Qian J., Sluss P.M., Rosner B.A., Hankinson S.E. (2013). A 20-year prospective study of plasma prolactin as a risk marker of breast cancer development. Cancer Res..

[59] Berinder K., Akre O., Granath F., Hulting A.L. (2011). Cancer risk in hyperprolactinemia patients: A population-based cohort study. Eur. J. Endocrinol..

[60] Dekkers O.M., Romijn J.A., de Boer A., Vandenbroucke J.P. (2010). The risk for breast cancer is not evidently increased in women with hyperprolactinemia. Pituitary.

[61] Vonderhaar B.K., Neville M.C., Daniel C.W. (1987). Prolactin: Transport, Function, and Receptors in Mammary Gland Development and Differentiation. The Mammary Gland.

[62] Davies T., Marians R., Latif R. (2002). The TSH receptor reveals itself. J. Clin. Investig..

[63] De Lloyd A., Bursell J., Gregory J.W., Rees D.A., Ludgate M. (2010). TSH receptor activation and body composition. J. Endocrinol..

[64] Slominski A., Wortsman J., Kohn L., Ain K.B., Venkataraman G.M., Pisarchick A., Chung J.H., Giuliani C., Thornton M., Slugocki G. (2002). Expression of hypothalamic-pituitary-thyroid axis related genes in the human skin. J. Investig. Dermatol..

[65] Cianfarani F., Baldini E., Cavalli A., Marchioni E., Lembo L., Teson M., Persechino S., Zambruno G., Ulisse S., Odorisio T. (2010). TSH receptor and thyroid-specific gene expression in human skin. J. Investig. Dermatol..

[66] Shi X.Z., Xue L., Jin X., Xu P., Jia S., Shen H.M. (2016). Different expression of sodium-iodide importer (NIS) between lactating breast and thyroid tissues may be due to structural difference of thyroid-stimulating hormone receptor (TSHR). J. Endocrinol. Investig..

[67] Govindaraj V., Yaduvanshi N.S., Krishnamachar H., Rao A.J. (2012). Expression of thyroid-stimulating hormone receptor, octamer-binding transcription factor 4, and intracisternal A particle-promoted polypeptide in human breast cancer tissues. Horm. Mol. Biol. Clin. Investig..

[68] Rivas M., Santisteban P. (2003). TSH-activated signaling pathways in thyroid tumorigenesis. Mol. Cell. Endocrinol..

[69] Medina D.L., Santisteban P. (2000). Thyrotropin-dependent proliferation on in vitro rat thyroid cell systems. Eur. J. Endocrinol..

[70] McLeod D.S., Watters K.F., Carpenter A.D., Ladenson P.W., Cooper D.S., Ding E.L. (2012). Thyrotropin and thyroid cancer diagnosis: A systematic review and dose-response meta-analysis. J. Clin. Endocrinol. Metab..

[71] Sorrenti S., Carbotta G., Di Matteo F.M., Catania A., Pironi D., Tartaglia F., Tarroni D., Gagliardi F., Tripodi D., Watanabe M. (2020). Evaluation of clinicopathological and molecular parameters on disease recurrence of papillary thyroid cancer patient: A retrospective observational study. Cancers.

[72] Ulisse S., Baldini E., Lauro A., Pironi D., Tripodi D., Lori E., Ferent I.C., Amabile M.I., Catania A., Di Matteo F.M. (2021). Papillary thyroid cancer prognosis: An evolving field. Cancers.

[73] Cooper D.S., Doherty G.M., Haugen B.R., Kloos R.T., Lee S.L., Mandel S.J., Mazzaferri E.L., McIver B., Pacini F., Schlumberger M. (2009). Revised american thyroid association management guidelines for patients with thyroid nodules and differentiated thyroid cancer. Thyroid.

[74] Ellerhorst J.A., Sendi-Naderi A., Johnson M.K., Cooke C.P., Dang S.M., Diwan A.H. (2006). Human melanoma cells express functional receptors for thyroid-stimulating hormone. Endocr. Relat. Cancer.

[75] Szychta P., Szychta W., Lewiński A., Karbownik-Lewińska M. (2015). Co-existence of chronic non-communicable diseases and common neoplasms among 2462 endocrine adult inpatients—A retrospective analysis. Ann. Agric. Environ. Med..

[76] Chen Y.K., Lin C.L., Chang Y.J., Cheng F.T., Peng C.L., Sung F.C., Cheng Y.H., Kao C.H. (2013). Cancer risk in patients with Graves’ disease: A nationwide cohort study. Thyroid.

[77] Szychta P., Szychta W., Gesing A., Lewiński A., Karbownik-Lewińska M. (2013). TSH receptor antibodies have predictive value for breast cancer—Retrospective analysis. Thyroid Res..

[78] Siegler J.E., Li X., Jones S.D., Kandil E. (2012). Early-onset breast cancer in a woman with Graves’ disease. Int. J. Clin. Exp. Med..

[79] Prinzi N., Baldini E., Sorrenti S., De Vito C., Tuccilli C., Catania A., Carbotta S., Mocini R., Coccaro C., Nesca A. (2014). Prevalence of breast cancer in thyroid diseases: Results of a cross-sectional study of 3921 patients. Breast Cancer Res. Treat..

[80] Greenspan F.S., Greenspan F.S., Gardner D.G. (2004). The Thyroid Gland. Basic & Clinical Endocrinology.

[81] Cheng S.Y., Leonard J.L., Davis P.J. (2010). Molecular aspects of thyroid hormone actions. Endocr. Rev..

[82] Brent G.A. (2012). Mechanisms of thyroid hormone action. J. Clin. Investig..

[83] Visser T.J. (2013). Thyroid hormone transporters and resistance. Endocr. Dev..

[84] Chin W.W., Parker M.G. (1991). Nuclear Thyroid Hormone Receptors. Nuclear Hormone Receptor.

[85] Ortiga-Carvalho T.M., Sidhaye A.R., Wondisford F.E. (2014). Thyroid hormone receptors and resistance to thyroid hormone disorders. Nat. Rev. Endocrinol..

[86] Lazar M.A., Chin W.W. (1990). Nuclear thyroid hormone receptors. J. Clin. Investig..

[87] Sap J., Munoz A., Damm K., Goldberg Y., Ghysdael J., Leutz A., Beug H., Vennström B. (1986). The c-erbA protein is a high-affinity receptor for thyroid hormone. Nature.

[88] Weinberger C., Thompson C.C., Ong E.S., Lebo R., Gruol D.J., Evans R.M. (1986). The c-erbA gene encodes a thyroid hormone receptor. Nature.

[89] Chassande O., Fraichard A., Gauthier K., Flamant F., Legrand C., Savatier P., Laudet V., Samarut J. (1997). Identification of transcripts initiated from an internal promoter in the c-erbA alpha locus that encode inhibitors of retinoic acid receptor-alpha and triiodothyronine receptor activities. Mol. Endocrinol..

[90] Gauthier K., Chassande O., Plateroti M., Roux J.P., Legrand C., Pain B., Rousset B., Weiss R., Trouillas J., Samarut J. (1999). Different functions for the thyroid hormone receptors TRalpha and TRbeta in the control of thyroid hormone production and post-natal development. EMBO J..

[91] Lin H.Y., Chin Y.T., Yang Y.C.S.H., Lai H.Y., Whang-Peng J., Liu L.F., Tang H.Y., Davis P.J. (2016). Thyroid hormone, cancer, and apoptosis. Compr. Physiol..

[92] Lin H.Y., Sun M., Tang H.Y., Lin C., Luidens M.K., Mousa S.A., Incerpi S., Drusano G.L., Davis F.B., Davis P.J. (2009). L-Thyroxine vs. 3,5,3’-triiodo-L-thyronine and cell proliferation: Activation of mitogen-activated protein kinase and phosphatidylinositol 3-kinase. Am. J. Physiol. Cell. Physiol..

[93] De Sibio M.T., De Oliveira M., Moretto F.C., Olimpio R.M., Conde S.J., Luvizon A.C., Nogueira C.R. (2014). Triiodothyronine and breast cancer. World J. Clin. Oncol..

[94] Brisken C., Ataca D. (2015). Endocrine hormones and local signals during the development of the mouse mammary gland. Wiley Interdiscip. Rev. Dev. Biol..

[95] Sellitti D.F., Tseng Y.C., Latham K.R. (1983). Nuclear thyroid hormone receptors in C3H/HeN mouse mammary glands and spontaneous tumors. Cancer Res..

[96] Hovey R.C., Trott J.F., Vonderhaar B.K. (2002). Establishing a framework for the functional mammary gland: From endocrinology to morphology. J. Mammary Gland Biol. Neoplasia.

[97] Topper Y.J., Freeman C.S. (1980). Multiple hormone interactions in the developmental biology of the mammary gland. Physiol. Rev..

[98] Meites J., Kragt C.L. (1964). Effects of a pituitary homotransplant and thyroxine on body and mammary growth in immature hypophysectomized rats. Endocrinology.

[99] Bhattacharya A., Vonderhaar B.K. (1979). Thyroid hormone regulation of prolactin binding to mouse mammary glands. Biochem. Biophys. Res. Commun..

[100] Borellini F., Oka T. (1989). Growth control and differentiation in mammary epithelial cells. Environ. Health Perspect..

[101] Groner A.C., Brown M. (2017). Role of steroid receptor and coregulator mutations in hormone-dependent cancers. J. Clin. Investig..

[102] Tryfonidis K., Zardavas D., Katzenellenbogen B.S., Piccart M. (2016). Endocrine treatment in breast cancer: Cure, resistance and beyond. Cancer Treat. Rev..

[103] Murphy C.G., Dickler M.N. (2016). Endocrine resistance in hormone-responsive breast cancer: Mechanisms and therapeutic strategies. Endocr. Relat. Cancer.

[104] Castrellon A.B. (2017). Novel strategies to improve the endocrine therapy of breast cancer. Oncol. Rev..

[105] Cirocchi R., Amabile M.I., De Luca A., Frusone F., Tripodi D., Gentile P., Tabola R., Pironi D., Forte F., Monti M. (2021). New classifications of axillary lymph nodes and their anatomical-clinical correlations in breast surgery. World J. Surg. Oncol..

[106] Amabile M.I., De Luca A., Tripodi D., D’Alberti E., Melcarne R., Imbimbo G., Picconi O., D’Andrea V., Vergine M., Sorrenti S. (2021). Effects of inositol hexaphosphate and myo-inositol administration in breast cancer patients during adjuvant chemotherapy. J. Pers. Med..

[107] Amabile M.I., Frusone F., De Luca A., Tripodi D., Imbimbo G., Lai S., D’Andrea V., Sorrenti S., Molfino A. (2020). Locoregional surgery in metastatic breast cancer: Do concomitant metabolic aspects have a role on the management and prognosis in this setting?. J. Pers. Med..

[108] Ulisse S., Tata J.R. (1994). Thyroid hormone and glucocorticoid independently regulate the expression of estrogen receptor in male Xenopus liver cells. Mol. Cell. Endocrinol..

[109] Alarid E.T., Preisler-Mashek M.T., Solodin N.M. (2003). Thyroid hormone is an inhibitor of estrogen-induced degradation of estrogen receptor-alpha protein: Estrogen-dependent proteolysis is not essential for receptor transactivation function in the pituitary. Endocrinology.

[110] Glass C.K., Holoway J.M. (1990). Regulation of gene expression by the thyroid hormone receptor. Biochim. Biophys. Acta.

[111] Tang H.Y., Lin H.Y., Zhang S., Davis F.B., Davis P.J. (2004). Thyroid hormone causes mitogen-activated protein kinase-dependent phosphorylation of the nuclear estrogen receptor. Endocrinology.

[112] Hercbergs A., Mousa S.A., Leinung M., Lin H.Y., Davis P.J. (2018). Thyroid hormone in the clinic and breast cancer. Horm. Cancer.

[113] Hammes S.R., Davis P.J. (2015). Overlapping nongenomic and genomic actions of thyroid hormone and steroids. Best Pract. Res. Clin. Endocrinol. Metab..

[114] Glinskii A.B., Glinsky G.V., Lin H.Y., Tang H.Y., Sun M., Davis F.B., Luidens M.K., Mousa S.A., Hercbergs A.H., Davis P.J. (2009). Modification of survival pathway gene expression in human breast cancer cells by tetraiodothyroacetic acid (tetrac). Cell Cycle.

[115] Davis P.J., Glinsky G.V., Lin H.Y., Leith J.T., Hercbergs A., Tang H.Y., Ashur-Fabian O., Incerpi S., Mousa S.A. (2015). Cancer cell gene expression modulated from plasma membrane integrin αvβ3 by thyroid hormone and nanoparticulate tetrac. Front. Endocrinol..

[116] Krashin E., Piekiełko-Witkowska A., Ellis M., Ashur-Fabian O. (2019). Thyroid hormones and cancer: A comprehensive review of preclinical and clinical studies. Front. Endocrinol..

[117] Suhane S., Ramanujan V.K. (2011). Thyroid hormone differentially modulates Warburg phenotype in breast cancer cells. Biochem. Biophys. Res. Commun..

[118] Flamini M.I., Uzair I., Pennacchio G.E., Neira F.J., Mondaca J.M., Cuello-Carrión F.D., Jahn G.A., Simoncini T., Sanchez A.M. (2017). Thyroid hormone controls breast cancer cell movement via integrin αV/β3/SRC/FAK/PI3-kinases. Horm. Cancer.

[119] Lin H.Y., Chin Y.T., Nana A.W., Shih Y.J., Lai H.Y., Tang H.Y., Leinung M., Mousa S.A., Davis P.J. (2016). Actions of l-thyroxine and Nano-diamino-tetrac (Nanotetrac) on PD-L1 in cancer cells. Steroids.

[120] Angelousi A., Diamanti-Kandarakis E., Zapanti E., Nonni A., Ktenas E., Mantzou A., Kontzoglou K., Kouraklis G. (2017). Is there an association between thyroid function abnormalities and breast cancer?. Arch. Endocrinol. Metab..

[121] Khan S.R., Chaker L., Ruiter R., Aerts J.G., Hofman A., Dehghan A., Franco O.H., Stricker B.H., Peeters R.P. (2016). Thyroid function and cancer risk: The rotterdam study. J. Clin. Endocrinol. Metab..

[122] Journy N.M.Y., Bernier M.O., Doody M.M., Alexander B.H., Linet M.S., Kitahara C.M. (2017). Hyperthyroidism, hypothyroidism, and cause-specific mortality in a large cohort of women. Thyroid.

[123] Cristofanilli M., Yamamura Y., Kau S.W., Bevers T., Strom S., Patangan M., Hsu L., Krishnamurthy S., Theriault R.L., Hortobagyi G.N. (2005). Thyroid hormone and breast carcinoma. Primary hypothyroidism is associated with a reduced incidence of primary breast carcinoma. Cancer.

[124] Søgaard M., Farkas D.K., Ehrenstein V., Jørgensen J.O., Dekkers O.M., Sørensen H.T. (2016). Hypothyroidism and hyperthyroidism and breast cancer risk: A nationwide cohort study. Eur. J. Endocrinol..

[125] Tosovic A., Bondeson A.G., Bondeson L., Ericsson U.B., Malm J., Manjer J. (2010). Prospectively measured triiodothyronine levels are positively associated with breast cancer risk in postmenopausal women. Breast Cancer Res..

[126] Shi X.Z., Jin X., Xu P., Shen H.M. (2014). Relationship between breast cancer and levels of serum thyroid hormones and antibodies: A meta-analysis. Asian Pac. J. Cancer Prev..

[127] Nisman B., Allweis T.M., Carmon E., Kadouri L., Maly B., Maimon O., Meierovich A., Peretz T. (2020). Thyroid hormones, silencing mediator for retinoid and thyroid receptors and prognosis in primary breast cancer. Anticancer Res..

[128] Kim E.Y., Chang Y., Lee K.H., Yun J.S., Park Y.L., Park C.H., Ahn J., Shin H., Ryu S. (2019). Serum concentration of thyroid hormones in abnormal and euthyroid ranges and breast cancer risk: A cohort study. Int. J. Cancer.

[129] Ortega-Olvera C., Ulloa-Aguirre A., Ángeles-Llerenas A., Mainero-Ratchelous F.E., González-Acevedo C.E., Hernández-Blanco M.L., Ziv E., Avilés-Santa L., Pérez-Rodríguez E., Torres-Mejía G. (2018). Thyroid hormones and breast cancer association according to menopausal status and body mass index. Breast Cancer Res..

[130] Rasool M., Naseer M.I., Zaigham K., Malik A., Riaz N., Alam R., Manan A., Sheikh I.A., Asif M. (2014). Comparative study of alterations in tri-iodothyronine (T3) and thyroxine (T4) hormone levels in breast and ovarian cancer. Pak. J. Med. Sci..

[131] Tosovic A., Bondeson A.G., Bondeson L., Ericsson U.B., Manjer J. (2014). T3 levels in relation to prognostic factors in breast cancer: A population-based prospective cohort study. BMC Cancer.

[132] Glushakov R.I., Proshin S.N., Tapil’Skaya N.I. (2013). The incidence of breast tumor during experimental hyperthyroidism. Bull. Exp. Biol. Med..

[133] Tran T.V.T., Maringe C., Majano S.B., Rachet B., Boutron-Rualt M.C., Journy N. (2021). Thyroid dysfunction and breast cancer risk among women in the UK biobank cohort. Cancer Med..

[134] Sterle H.A., Hildebrandt X., Valenzuela Álvarez M., Paulazo M.A., Gutierrez L.M., Klecha A.J., Cayrol F., Díaz Flaqué M.C., Rosemblit C., Barreiro Arcos M.L. (2021). Thyroid status regulates the tumor microenvironment delineating breast cancer fate. Endocr. Relat. Cancer.

[135] Weng C.H., Okawa E.R., Roberts M.B., Park S.K., Umbricht C.B., Manson J.E., Eaton C.B. (2020). Breast cancer risk in postmenopausal women with medical history of thyroid disorder in the women’s health initiative. Thyroid.

[136] Yang H., Holowko N., Grassmann F., Eriksson M., Hall P., Czene K. (2020). Hyperthyroidism is associated with breast cancer risk and mammographic and genetic risk predictors. BMC Med..

[137] Yuan S., Kar S., Vithayathil M., Carter P., Mason A.M., Burgess S., Larsson S.C. (2020). Causal associations of thyroid function and dysfunction with overall, breast and thyroid cancer: A two-sample Mendelian randomization study. Int. J. Cancer.

[138] Tran T.V., Kitahara C.M., de Vathaire F., Boutron-Ruault M.C., Journy N. (2020). Thyroid dysfunction and cancer incidence: A systematic review and meta-analysis. Endocr. Relat. Cancer.

[139] Weng C.H., Chen Y.H., Lin C.H., Luo X., Lin T.H. (2018). Thyroid disorders and breast cancer risk in Asian population: A nationwide population-based case-control study in Taiwan. BMJ Open.

[140] Cordel E., Reix N., Molière S., Mathelin C. (2018). Hyperthyroidism and breast cancer: Is there a link?. Gynecol. Obstet. Fertil. Senol..

[141] Ferreira E., da Silva A.E., Serakides R., Gomes M.G., Cassali G.D. (2007). Ehrlich tumor as model to study artificial hyperthyroidism influence on breast cancer. Pathol. Res. Pract..

[142] Bergh J.J., Lin H.Y., Lansing L., Mohamed S.N., Davis F.B., Mousa S., Davis P.J. (2005). Integrin alphaVbeta3 contains a cell surface receptor site for thyroid hormone that is linked to activation of mitogen-activated protein kinase and induction of angiogenesis. Endocrinology.

[143] Hercbergs A., Johnson R.E., Ashur-Fabian O., Garfield D.H., Davis P.J. (2015). Medically induced euthyroid hypothyroxinemia may extend survival in compassionate need cancer patients: An observational study. Oncologist.

[144] Kim W.G., Cheng S.Y. (2013). Thyroid hormone receptors and cancer. Biochim. Biophys. Acta.

[145] Silva J.M., Domínguez G., González-Sancho J.M., García J.M., Silva J., García-Andrade C., Navarro A., Muñoz A., Bonilla F. (2002). Expression of thyroid hormone receptor/erbA genes is altered in human breast cancer. Oncogene.

[146] Saraiva P.P., Figueiredo N.B., Padovani C.R., Brentani M.M., Nogueira C.R. (2005). Profile of thyroid hormones in breast cancer patients. Braz. J. Med. Biol. Res..

[147] Chen L.C., Matsumura K., Deng G., Kurisu W., Ljung B.M., Lerman M.I., Waldman F.M., Smith H.S. (1994). Deletion of two separate regions on chromosome 3p in breast cancers. Cancer Res..

[148] Futreal P.A., Söderkvist P., Marks J.R., Iglehart J.D., Cochran C., Barrett J.C., Wiseman R.W. (1992). Detection of frequent allelic loss on proximal chromosome 17q in sporadic breast carcinoma using microsatellite length polymorphisms. Cancer Res..

[149] Ling Y., Xu X., Hao J., Ling X., Du X., Liu X., Zhao X. (2010). Aberrant methylation of the THRB gene in tissue and plasma of breast cancer patients. Cancer Genet. Cytogenet..

[150] Li Z., Meng Z.H., Chandrasekaran R., Kuo W.L., Collins C.C., Gray J.W., Dairkee S.H. (2002). Biallelic inactivation of the thyroid hormone receptor beta1 gene in early stage breast cancer. Cancer Res..

[151] Ling Y., Ling X., Fan L., Wang Y., Li Q. (2015). Mutation analysis underlying the downregulation of the thyroid hormone receptor β1 gene in the Chinese breast cancer population. OncoTargets Ther..

[152] Park J.W., Zhao L., Cheng S.-Y. (2013). Inhibition of estrogen-dependent tumorigenesis by the thyroid hormone receptor b in xenograft models. Am. J. Cancer Res..

[153] Sarlis N.J., Gourgiotis L., Pucino F., Tolis G.J. (2002). Lack of association between Hashimoto thyroiditis and breast cancer: A quantitative research synthesis. Hormones.

[154] Prinzi N., Sorrenti S., Baldini E., De Vito C., Tuccilli C., Catania A., Coccaro C., Bianchini M., Nesca A., Grani G. (2015). Association of thyroid diseases with primary extra-thyroidal malignancies in women: Results of a cross-sectional study of 6386 patients. PLoS ONE.

[155] Smyth P.P., Shering S.G., Kilbane M.T., Murray M.J., McDermott E.W., Smith D.F., O’Higgins N.J. (1998). Serum thyroid peroxidase autoantibodies, thyroid volume, and outcome in breast carcinoma. J. Clin. Endocrinol. Metab..

[156] Weijl N.I., Van der Harst D., Brand A., Kooy Y., Van Luxemburg S., Schroder J., Lentjes E., Van Rood J.J., Cleton F.J., Osanto S. (1993). Hypothyroidism during immunotherapy with interleukin-2 is associated with antithyroid antibodies and response to treatment. J. Clin. Oncol..

[157] Franzke A., Peest D., Probst-Kepper M., Buer J., Kirchner G.I., Brabant G., Kirchner H., Ganser A., Atzpodien J. (1999). Autoimmunity resulting from cytokine treatment predicts long-term survival in patients with metastatic renal cell cancer. J. Clin. Oncol..

[158] Smyth P.P.A. (2000). Autoimmune thyroid disease and breast cancer: A chance association?. J. Endocrinol. Investig..

[159] Rodien P., Madec A.M., Ruf J., Rajas F., Bornet H., Carayon P., Orgiazzi J. (1996). Antibody-dependent cell-mediated cytotoxicity in autoimmune thyroid disease: Relationship to antithyroperoxidase antibodies. J. Clin. Endocrinol. Metab..

[160] Spitzweg C., Joba W., Eisenmenger W., Heufelder A.E. (1998). Analysis of human sodium iodide symporter gene expression in extrathyroidal tissues and cloning of its complementary deoxyribonucleic acids from salivary gland, mammary gland, and gastric mucosa. J. Clin. Endocrinol. Metab..

[161] Graceffa G., Scerrino G., Militello G., Laise I., Randisi B., Melfa G., Orlando G., Mazzola S., Cipolla C., Cocorullo G. (2021). Breast cancer in previously thyroidectomized patients: Which thyroid disorders are a risk factor?. Future Sci. OA.

[162] Bach L., Kostev K., Schiffmann L., Kalder M. (2020). Association between thyroid gland diseases and breast cancer: A case-control study. Breast Cancer Res. Treat..

[163] Dobrinja C., Scomersi S., Giudici F., Vallon G., Lanzaro A., Troian M., Bonazza D., Romano A., Zanconati F., de Manzini N. (2019). Association between benign thyroid disease and breast cancer: A single center experience. BMC Endocr. Disord..

[164] Rahman S., Archana A., Jan A.T., Dutta D., Shankar A., Kim J., Minakshi R. (2019). Molecular insights into the relationship between autoimmune thyroid diseases and breast cancer: A critical perspective on autoimmunity and ER stress. Front. Immunol..

